# Febrile seizures after 2009 influenza A (H1N1) vaccination and infection: a nationwide registry-based study

**DOI:** 10.1186/s12879-015-1263-7

**Published:** 2015-11-09

**Authors:** Inger Johanne Bakken, Kari Modalsli Aaberg, Sara Ghaderi, Nina Gunnes, Lill Trogstad, Per Magnus, Siri Eldevik Håberg

**Affiliations:** Norwegian Institute of Public Health, Nydalen, PO Box 4404, Oslo, N-0403 Norway; National Centre for Epilepsy, Oslo University Hospital, Oslo, Norway

**Keywords:** Vaccine safety, Febrile seizures, Influenza A (H1N1) vaccination, Influenza A (H1N1) infection

## Abstract

**Background:**

During the 2009 influenza A (H1N1) pandemic, a monovalent pandemic strain vaccine containing the oil-in-water adjuvant AS03 (Pandemrix®) was offered to the Norwegian population. The coverage among children reached 54 %. Our aim was to estimate the risk of febrile seizure in children after exposure to pandemic influenza vaccination or infection.

**Methods:**

The study population comprised 226,889 children born 2006–2009 resident in Norway per October 1st, 2009. Febrile seizure episodes were defined by emergency hospital admissions / emergency outpatient hospital care with International Classification of Diseases, Version 10, codes R56.0 or R56.8. The self-controlled case series method was applied to estimate incidence rate ratios (IRRs) in pre-defined risk periods compared to the background period. The total observation window was ± 180 days from exposure day. Among 113,068 vaccinated children, 656 (0.6 %) had at least one febrile seizure episode.

**Results:**

The IRR of febrile seizures 1–3 days after vaccination was 2.00 (95 % confidence interval [CI]: 1.15–3.51). In the period 4–7 days after vaccination, no increased risk was observed. Among the 8172 children diagnosed with pandemic influenza, 84 (1.0 %) had at least one febrile seizure episode. The IRR of febrile seizures on the same day as a diagnosis of influenza was 116.70 (95 % CI: 62.81–216.90). In the period 1–3 days after a diagnosis of influenza, a tenfold increased risk was observed (IRR 10.12, 95 % CI: 3.82 – 26.82).

**Conclusions:**

In this large population-based study with precise timing of exposures and outcomes, we found a twofold increased risk of febrile seizures 1–3 days after pandemic influenza vaccination. However, we found that pandemic influenza infection was associated with a much stronger increase in risk of febrile seizures.

## Background

Febrile seizure is estimated to occur in 2 to 5 % of all children 6 through 60 months of age, and is defined as seizures accompanied by fever (≥38 °C) without central nervous system infection [1]. Several viruses, such as human herpesvirus 6 and influenza viruses, have been associated with increased risk of febrile seizures [2–6].

Some vaccines, including influenza vaccines, have also been reported to increase the risk of febrile seizures [7–12]. Indeed, vaccine administration is the second leading cause of febrile seizures [13]. The main modulator of the condition is the seizure threshold, which varies strongly between individuals and is influenced by age, maturation, and genetic predisposition [4, 14].

During the 2009 influenza A (H1N1) pandemic, a monovalent pandemic strain vaccine containing the oil-in-water adjuvant AS03 (Pandemrix®) was offered free of charge to the Norwegian population, with the national vaccination coverage among children reaching 54 % [15].

The aim of the present study was to investigate whether there was an increased risk of febrile seizures in young children following pandemic influenza vaccination or infection, using a population-based design with information on exposures and outcome from national health registries.

## Methods

The study was approved by the Regional Committee for Medical and Health Research Ethics, South-East Norway.

### The influenza A (H1N1) pandemic in Norway

**Fig. 1:**
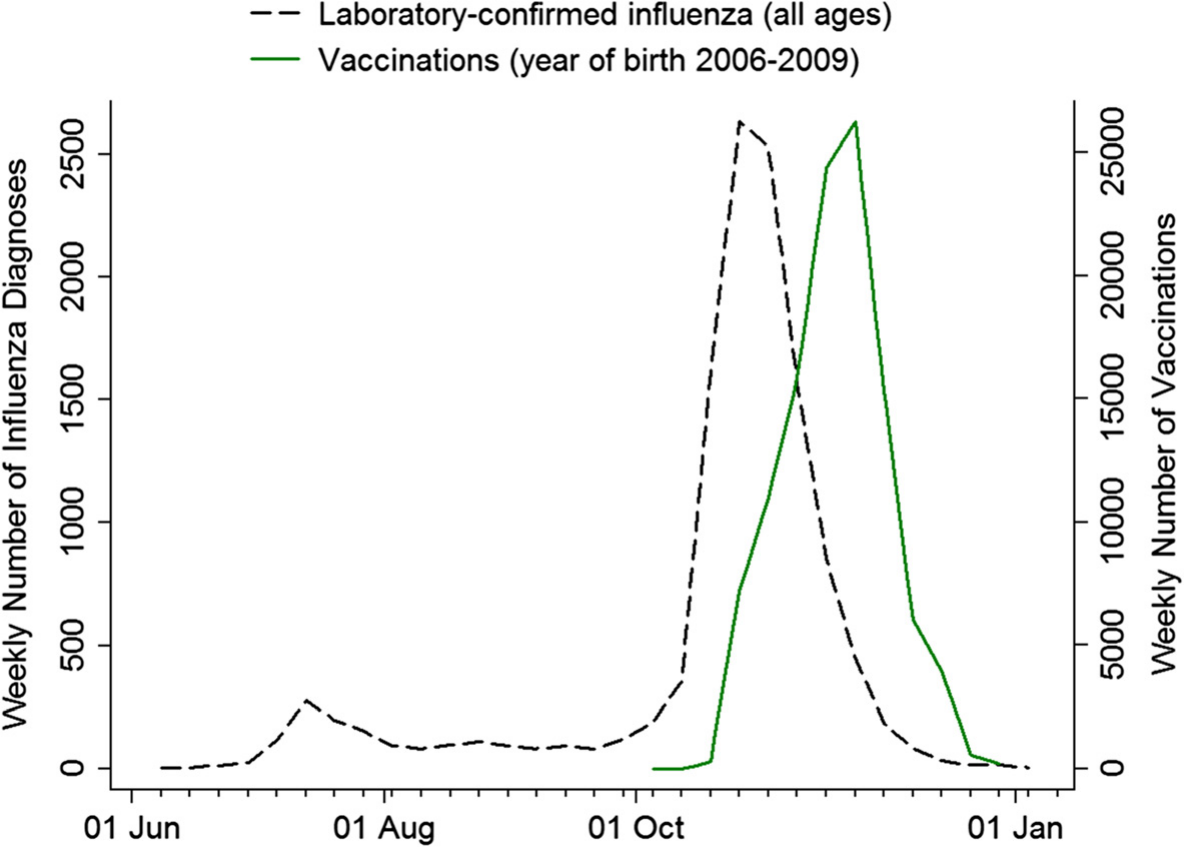
Timing of the influenza pandemic in Norway illustrated by number of laboratory-confirmed cases per week of influenza A (H1N1) (all ages) and the timing of the vaccination campaign given by the number of pandemic influenza vaccinations per week in children born in 2006–2009

In Norway, the majority of laboratory-confirmed cases of influenza A (H1N1) infection were registered during the main pandemic wave from October 1^st^ through December 31^st^, 2009 (Fig. 1). From October 19^th^, 2009, two vaccines became available: Pandemrix® (GlaxoSmithKline), which contained the squalene-based adjuvant AS03, and Celvapan® (Baxter), which did not. Pandemrix® was the recommended vaccine for all ages, while Celvapan® was only offered to people with severe egg allergy. The vaccination period overlapped with the main period of the pandemic wave. However, a small number of vaccine doses were administered after the main pandemic wave (Fig. 1).

### Study population

The study population comprised all Norwegian children born between January 1^st^, 2006, and October 1^st^, 2009 (i.e., children up to 45 months of age), who were registered in the National Registry as residents of Norway as of October 1^st^, 2009 (n = 227,133). We excluded children vaccinated with Celvapan® but not with Pandemrix® (n = 61) and children registered as vaccinated but lacking information on the date of vaccination (n = 183). A total of 226,889 children were eligible for the study.

### Data sources

All individuals were assigned a study allocation number based on the 11-digit personal identification number unique to all Norwegian citizens and migrants with legal residence in Norway. In addition to information from the national population registry, we used information from the Norwegian Directorate of Health, the Norwegian Surveillance System for Communicable Diseases, the Norwegian Immunisation Register, and the Norwegian Patient Register.

### Exposures

The exposures under study were pandemic influenza vaccination with Pandemrix® and pandemic influenza infection.

The Norwegian Immunisation Register provided information on the two influenza vaccines that were used in Norway against the H1N1 strain. For the general population, a single dose of Pandemrix® was recommended by the Norwegian Institute of Public Health. All vaccinations from October 19^th^, 2009, until the early months of 2010 were included in the analyses.

Information on influenza during the pandemic was obtained from the Norwegian Directorate of Health, where information on all consultations in primary health care and emergency outpatient clinics is reported for reimbursement purposes. Reporting of the personal identification number, the date of consultation, and International Classification of Primary Care, Second Edition (ICPC-2) codes is mandatory. The ICPC-2 code for influenza-like illness (R80) was taken as a measure of H1N1 infection when the diagnosis was assigned during the main pandemic wave (October 1^st^ through December 31^st^, 2009). In addition, we had access to information on positive antigenic tests for H1N1 infection from microbiology laboratories, as reported to the Norwegian Surveillance System for Communicable Diseases. These tests are highly specific, and we therefore also included such records from outside the main pandemic wave. However, the majority (91.3 %) of laboratory-confirmed influenza A (H1N1) infections were reported during the main pandemic wave.

### Outcome

Information on seizure episodes, defined as emergency hospitalization or emergency outpatient care with a registration of International Classification of Diseases, Version 10 (ICD-10) code R56.0 (“Febrile convulsions”) or R56.8 (“Other and unspecified convulsions”), was obtained from the Norwegian Patient Register. This registry holds data from all Norwegian hospitals, and reporting is mandatory and linked to the reimbursement system. Personal identification numbers have been reported from January 1^st^, 2008, onwards. Diagnoses are reported as ICD-10 codes. We used the day of admission as the seizure date in all analyses.

### Statistical analysis

We applied a self-controlled case series (SCCS) method to estimate the incidence rate ratio (IRR) of febrile seizures in various risk periods following influenza vaccination and influenza infection compared to the background period. This method eliminates time-independent confounding [16, 17] . Person-time and events for each individual were stratified by age (one-year bands), calendar period (January – March, April – August, and September – December), and risk period (background, day of vaccination or influenza diagnosis, and 1–3 and 4–7 days after vaccination or influenza diagnosis). In the analysis of vaccination effect, a pre-vaccination period of two weeks was analyzed separately to allow for delayed vaccination due to febrile convulsion. In the analysis of febrile seizures after a diagnosis of influenza, we also took a pre-exposure risk period out of the background period in order to exclude follow-up appointments in general practice for children who had been hospitalized for febrile seizures. For each individual, the observation period was restricted to a period starting 180 days prior to exposure or on the day of birth (whichever came last) and ending 180 days after exposure or on the day of emigration or death (whichever came first). Thus, each individual could contribute with a maximum of 360 observation days. IRR estimates, adjusted for age and calendar period, were obtained by using conditional Poisson regression.

In sensitivity analyses, we first repeated all analyses excluding outpatients and also applied a more strict definition of febrile seizures by using ICD-10 code R56.0 only. In addition, we applied multiple-event Cox proportional-hazards regression with time-dependent covariates for the exposure variables, using calendar day as the time metric.

The Stata software package, Version 13.0 (StataCorp. 2013. *Stata Statistical Software: Release 11*. College Station, TX: StataCorp LP) was used for data analysis.

## Results

In Norway, pandemic influenza vaccination was offered from October 19^th^, 2009. Among the 226,889 children in this study, 113,068 (49.8 %) were vaccinated and 8172 (3.6 %) were diagnosed with pandemic influenza in primary care. Table 1 shows vaccination coverage and distribution of influenza diagnoses by sex and year of birth. Most children received the vaccine in the late half of the main pandemic wave (Fig. 1), and 98.1 % of all pandemic vaccine doses were given before January 1^st^, 2010.

**Table 1:** Characteristics of the study population, all children born in the period from January 1^st^, 2006, through October 1^st^, 2009, and resident in Norway as of October 1^st^, 2009^a^

	Number	Number vaccinated (%)	Number with influenza diagnosis (%)
Total	226,889 (100)	113,068 (49.8)	8172 (3.6)
Sex			
Male	116,460 (51.3)	58,067 (49.9)	4405 (3.8)
Female	110,429 (48. 7)	55,001 (49.8)	3767 (3.4)
Year of birth			
2006	60,521 (26.7)	33,238 (54.9)	2489 (4.1)
2007	59,833 (26.4)	33,106 (55.3)	2328 (3.9)
2008	61,136 (27.0)	32,330 (52.9)	2423 (4.0)
2009	45,399 (20.0)	14,394 (31.7)	932 (2.1)

^a^Excluding 61 children vaccinated with Celvapan only® and 183 children with missing date of vaccination with Pandemrix®

**Fig. 2:**
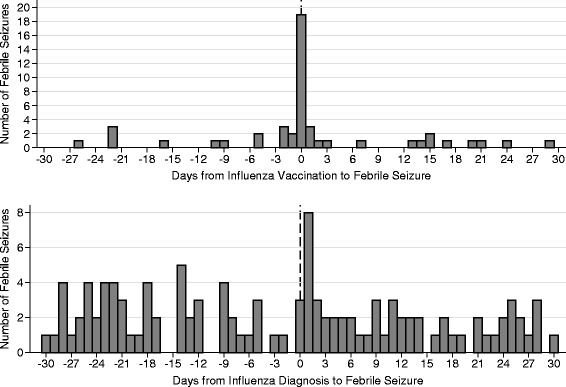
Number of days from influenza vaccination (*top panel*) and influenza infection (*lower panel*) to episode of febrile seizure

A total of 656 children had at least one febrile seizure episode in the period starting 180 days before vaccination and ending 180 days after vaccination (785 episodes in total). A plot of the number of daily febrile seizure episodes in a time period starting 30 days before and ending 30 days after day of vaccination suggests an excess of cases the first few days after vaccination (Fig. 2, top panel). A total of 23 cases were registered during the first week, corresponding to a total incidence of 23/113,068 or 20 per 100,000 vaccinated children. Results from the SCCS analyses showed an increased risk in the period 1–3 days following vaccination (IRR: 2.00, 95 % confidence interval [CI]: 1.15–3.51) (Table 2).

**Table 2:** Incidence rate ratio (IRR) estimates, with associated 95 % confidence intervals (CIs), of febrile seizure in relation to the timing of influenza vaccination and infection

Exposure	Period	No. of person-days at risk	No. of events	Incidence rate (per 100 person-days)	IRR^a^ (95 % CI)
	Background period^d^	976,010	739	0.08	1
	2 weeks before vaccination day	40,320	23	0.06	0.76 (0.50–1.43)
Vaccination^b^	Day of vaccination	2880	3	0.10	1.39 (0.46–4.35)
	1–3 days after vaccination	8640	13	0.15	2.00 (1.15–3.51)
	4–7 days after vaccination	11,520	7	0.06	0.81 (0.38 – 1.73)
	Background period	95,724	63	0.07	1
	2 weeks before influenza diagnosis	3962	9	0.23	3.96 (1.81–8.65)
Influenza^c^	Day of influenza diagnosis	283	19	6.71	116.70 (62.81–216.90)
	1–3 days after influenza diagnosis	849	5	0.59	10.12 (3.82–26.82)
	4–7 days after influenza diagnosis	1132	1	0.09	1.50 (0.20–11.17)

^a^Adjusted for calendar period (January–March, April–August, and September–December) and age in one-year bands

^b^Results from self-controlled case series analysis, data from 656 Norwegian children born in 2006–2009 vaccinated with Pandemrix® and having 785 febrile seizure episodes during the total observation period (starting 180 days prior to vaccination or on day of birth [whichever came last], and ending 180 days after exposure or on the day of emigration or death [whichever came first])

^c^Results from self-controlled case series analysis, data from 84 Norwegian children born in 2006–2009 diagnosed with pandemic influenza and having 97 seizures episodes during the total observation period (starting 180 days prior to vaccination or on day of birth [whichever came last], and ending 180 days after exposure or on the day of emigration or death [whichever came first])

^d^All other parts of the observation period

Overall, 84 children had at least one febrile seizure episode in the period starting 180 days before a diagnosis of pandemic influenza and ending 180 days after the diagnosis (97 episodes in total). There was a clear excess of specialist health care treatment for febrile seizures on the day of having an influenza diagnosis in primary health care (Fig. 2, lower panel). A total of 25 cases were registered during the first week, corresponding to a total incidence of 25/8127 or 308 per 100,000 children diagnosed with pandemic influenza. Results from the SCCS analyses showed a strong association between influenza infection and febrile seizures, especially on the day of diagnosis (IRR: 116.70, 95 % CI: 62.81 – 216.90). A tenfold increased risk was observed in the period 1–3 days after influenza diagnosis (Table 2). Among the children with febrile seizure during the first week following a diagnosis of pandemic influenza, one child had also been vaccinated during the same week. No other children contributed to the number of events in the week after exposure in both analyses shown in Table 2.

We finally conducted a series of sensitivity analyses. When outpatients were excluded the number of episodes eligible for the vaccination analyses was reduced from 785 to 567, while the number of episodes eligible for the influenza analyses was reduced from 97 to 82. In these analyses we obtained an IRR for the period 1–3 days following vaccination of 2.73 (95 % CI: 1.51 – 4.99) and an IRR for the day of an influenza diagnosis of 130.2 (95 % CI: 64.82 – 261.30). We subsequently defined febrile seizures by ICD-10 code R56.0 only (thus excluding R56.8). In these analyses, we obtained an IRR for the period 1–3 days following vaccination of 1.79 (95 % CI: 0.92 – 3.49) (528 episodes in total) and an IRR for the day of an influenza diagnosis of 133.30 (95 % CI: 70.82 – 250.80) (77 episodes in total). Finally, we conducted multiple-event Cox proportional-hazards regression analyses treating the exposure variables as time-dependent covariates. In those models the hazard ratio (HR) for the period 1–3 days following vaccination was 2.01 (95 % CI: 1.09 – 4.42) while the HR for the day of an influenza diagnosis was 142.84 (95 % CI: 76.86 – 265.46).

## Discussion

This study showed a strong association between pandemic influenza infection and febrile seizures. Furthermore, we observed a slightly increased short-term risk of febrile seizures following pandemic vaccination.

The major strengths of the present study are the population-based design and the precise timing of exposures and outcome, which is crucial for SCCS analysis [16]. Information on exposures and outcome was prospectively collected from independent national data sources, minimizing selection and information bias.

As in most other registry-based studies, we had limited information on potential confounders. The use of a self-controlled method, however, eliminated time-independent confounding. We furthermore adjusted for calendar period and age, which are time-dependent variables [17]. By using the SCCS method, we could apply multiple risk periods [17], making us able to distinguish between the immediate risk and the delayed risk of febrile seizures following vaccination or influenza infection. As an additional approach, we applied multiple-event Cox proportional-hazards regression with time-dependent exposure variables, which gave similar relative risk estimates.

Since reporting of diagnoses from primary health care consultations is mandatory for reimbursement, it seems likely that the number of influenza diagnoses reported reflects the true number of patients with influenza symptoms seeking medical care. However, many children with influenza were probably not diagnosed in primary care. The proportion of the population with clinical symptoms during the pandemic wave has been estimated at approximately 20 % [18], while only 3.6 % of the children in our study received an influenza diagnosis in primary care. Our results are therefore likely to apply to more severe influenza infections.

As in other studies based on observational, routinely collected data, a limitation of the present study is the lack of validity testing of the outcome diagnosis. We restricted the outcome to emergency febrile seizure episodes, and our study population comprised young children only. By excluding non-emergency health care, we reduced the potential influence of control consultations and thereby limiting outcome misclassification.

In the present study, we found a total incidence of febrile seizure of 20 per 100,000 vaccinated children in the first week after vaccination, which is close to what was reported for the MMR1 vaccine (24 per 100,000 vaccinated children) in an Australian registry-based study [6]. We found an IRR of 2.0 for the period 1–3 days post-vaccination, which is close to the IRR of 2.4 reported in a study of trivalent inactivated influenza vaccination in a study from the United States [19].

In a study from the UK based on data from the General Practice Research Database including children below the age of 10 years, SCCS analysis indicated a slight increase in the risk of seizures on the day of vaccination with Pandemrix® [10]. This increase did, however, not reach statistical significance, although the study was similar in size to our study with respect to the number of outcome events. While we based the definition of the outcome on data reported directly from the hospitals and restricted it to emergency events, the definition in the UK study had to rely on text searches in hospital discharge letters. The broader definition of the outcome in the UK study might explain the difference between that study and ours, as the risk of misclassification of events probably is lower in the present study.

In an SCCS study based on national Swedish registries and including people of all ages, no association was found between Pandemrix® vaccination and risk of febrile seizures, as defined by admission to hospital or outpatient hospital care with ICD-10 codes R56.0 or R56.8 [20]. However, febrile seizures usually occur in children under the age of five years [1], and by including adults, the major proportion of events might be cases of unspecified convulsions (R56.8) in adults, which may have concealed any association in young children.

In the Nordic countries, large cohort studies can be performed by linkage of population-based registries, while research in most other countries has to rely on other designs. In a study from New York, USA, parents of children vaccinated with trivalent inactivated influenza vaccine and/or 13-valent pneumococcal vaccine were texted on the night of vaccination and the seven subsequent nights to report their child’s temperature [21]. The study showed that among children receiving both vaccines on the same day, the proportion with fever on the day of vaccination was high (37.6 %), whereas it was lower in the groups receiving only one vaccine (7.5–9.5 %). However, febrile seizures were not studied.

We observed a strong increase in the risk of emergency hospitalization for seizures on the day of receiving an influenza diagnosis. In a previous study from Denmark, national registry data were used to correlate the weekly number of hospital admissions for febrile seizures with the activity of influenza-like illness as monitored by a sentinel-surveillance system of selected general practitioners [22]. Using this approach, it was concluded that influenza contributed to 29–47 % of such admissions. Other studies have also found that influenza accounts for a substantial proportion of febrile seizure episodes [23, 24]. This is supported by the present results, showing a large increase in the risk of febrile seizures following a diagnosis of pandemic influenza. Also, a study from the US with data from the influenza seasons 2004–5 through 2008–9 and including children ≥ 6 months and < 5 years of age showed that influenza vaccination rates were low while influenza was an important cause for hospitalizations and emergency department visits [25]. Furthermore, influenza can be fatal in children [26], and the American Academy of Pediatrics recommends annual seasonal influenza vaccination for all people ≥ 6 months and older [27].

We have studied febrile seizures in young children after influenza vaccination and influenza infection. In general, uncomplicated febrile seizure is a benign condition, and it has not been found to be associated with increased mortality or later neurocognitive difficulties in children without any prior neurological or developmental disorders [1, 28–31]. The risk of seizures varies strongly between individuals [14], and when counselling parents about the risk of febrile seizures following vaccination, factors such as age, neurological or developmental conditions, and genetic predisposition should be taken into account. Furthermore, it is important to hold the risk of febrile seizures following vaccination up against the severity of the disease the vaccine is to prevent. In our study, we observed only a slight increase in the number of febrile seizures following vaccination with the adjuvanted vaccine Pandemrix® but a strong increase following influenza infection. Furthermore, in a large recent study from the US, no increased risk of febrile seizure was found in children after administration of inactivated influenza vaccine [32].

## Conclusions

The present study has shown that the risk of emergency hospitalization for febrile seizures was increased following pandemic influenza vaccination. This increase in risk was, however, of short duration and was small compared to the massive increase in risk following a diagnosis of pandemic influenza infection.

## Abbreviations

CI: Confidence interval
ICD-10: International Classification of Diseases, Version 10
ICPC-2: International Classification of Primary Care, Second Edition
IRR: Incidence rate ratio
SCCS: Self-controlled case series
